# Differences in total and regional body fat and their association with BMI in UK-born White and South Asian children: findings from the Born in Bradford birth cohort

**DOI:** 10.12688/wellcomeopenres.16659.1

**Published:** 2021-03-26

**Authors:** Gillian Santorelli, Jane West, Tiffany Yang, John Wright, Maria Bryant, D.A. Lawlor

**Affiliations:** 1 Bradford Institute for Health Research, Bradford, BD9 6RJ, UK; 2 Department of Health Sciences and the Hull York Medical School, York, YO10 5DD, UK; 3 MRC Integrative Epidemiology Unit, University of Bristol, Bristol, BS8 2BN, Bahamas; 4 Population Health Sciences, Bristol Medical School, University of Bristol, Bristol, BS8 2BN, UK

**Keywords:** Ethnicity, DXA, adiposity, BMI, fat mass

## Abstract

**Background**: Body mass index (BMI) is commonly used as a proxy to determine excess adiposity, though this may underestimate fat mass (FM) in individuals of South Asian (SA) heritage. SA tend to have greater central adiposity than white people, which is associated with a higher risk of cardiometabolic disease. In this cross-sectional study, we aimed to determine the differences in total and regional FM using Dual-energy X-ray absorptiometry (DXA), and to see if any differences in FM varied by BMI category in UK-born white and SA children aged ~9 years.

**Methods**: Anthropometric measurements and DXA scans were undertaken from 225 white and 269 SA children from the Born in Bradford cohort study. Linear regression was used to assess ethnic differences in total body fat percent and total and regional FM.

**Results**: Although the mean BMI was similar, compared to white children, the proportion of SA children who were overweight or obese was ~20% higher, they had a median of 2kg more total FM, and the proportion with > 35% total body fat (TBF) was 22% and 16% higher in boys and girls respectively. Mean TBF% was greater in each BMI category, as was truncal, android and gynoid FM, with the greatest differences between ethnic groups observed in the healthy and overweight categories.

**Conclusions**: Greater TBF% and total and regional FM in the healthy- and overweight BMI categories observed in SA children suggests they may be at greater risk of future cardiometabolic disease at a BMI level below obesity threshold. However, our sample size was small, and results may be influenced by selection bias and confounding; our findings need to be replicated in a larger study.

## Introduction

Greater adiposity has been shown to increase the risk of type 2 diabetes mellitus (T2DM) and cardiovascular disease (CVD) in adults ^1– 3^ and children ^4– 8^. Risk of cardiometabolic disease is greater among those of South Asian (SA) heritage, who for a given body mass index (BMI) have greater total and central adiposity and are more likely to be insulin resistant and have CVD risk factors compared to White European adults ^9– 11 ^and children ^12– 15^. Specifically, it is visceral fat mass (FM), which is located in the trunk, rather than subcutaneous FM, that has been consistently associated with a higher risk of cardiometabolic disease independently of total FM ^16^. Thus, android fat, which is characterised by truncal obesity, may confer a greater risk of morbidity whereas gynoid fat (i.e. in the gluteofemoral region), which is subcutaneous, has been shown to be potentially protective ^16^. Further, the android/gynoid FM ratio has been shown to be predictive of cardiometabolic dysregulation in both adults ^17^ and children ^18– 20^.

Excess adiposity is commonly determined using BMI. Though BMI is a weight-basead index that cannot distinguish between fat mass (FM) and lean mass (LM), it is highly correlated with both. However, using BMI alone may underestimate FM in people of SA heritage, which has led to suggestions that BMI cut-points be adjusted in Asian adults ^21^ and children ^15^.

Studies conducted using more advanced methods to accurately quantify adiposity using Dual-energy X-ray absorptiometry (DXA) in different UK ethnic groups have found that South Asian children have significantly more total body fat compared to white children ^15, 22^, but to our knowledge none have reported ethnic differences in regional FM.

The aims of this study were to (i) determine the magnitude and direction of any differences in total and regional FM measured using DXA scans, and (ii) explore whether any differences in total and regional FM vary by BMI category, in UK-born white and SA-origin children aged approximately 9 years.

## Methods

### Participants and setting

This cross-sectional study used data obtained from participants of the Born in Bradford (BiB) multi-ethnic pregnancy and birth cohort study, details of which have been described elsewhere ^23^. Briefly, 12,453 pregnant women across 13,733 pregnancies were recruited between March 2007 and December 2010, resulting in 13,858 births. In 2017, a follow-up study of BiB participants was launched ^24^, and recruitment is expected to continue to June 2021. Briefly, the main objectives were to (i) investigate the determinants of child social and emotional wellbeing, (ii) identify the determinants of healthy growth and of adiposity and cardiometabolic health, and (iii) investigate the determinants of cognitive and sensorimotor development. The minimum effect sizes detectable for a range of outcomes were calculated based on expected participation. Families were invited to participate via a letter which included information sheets, followed by a telephone call from a BiB researcher. Assessments were conducted on a mobile health research bus or, if preferable to participants, via home visits, appointments in community locations, or through telephone or postal questionnaires. The health research bus was loaned from the University of Birmingham between February 2017 to June 2018. It comprised a procedure room, sample room and scanner room, and was equipped with a DXA machine, benchtop refrigerated centrifuge, fridge, and freezer. As the DXA scans could only be conducted on the health research bus, it was at various times located in four different areas of the city to increase the catchment area.

### Ethical considerations

Ethical approval was granted by the National Health Service Health Research Authority Yorkshire and the Humber (Bradford Leeds) Research Ethics Committee (reference: 16/YH/0320). Informed consent for the data collection was provided by the child’s caregiver at recruitment to the BiB cohort study, but parents were informed that they could opt-out consent for measures collected in the follow-up study at their assessment appointment.

### Scan acquisition and variables

During the loan period of the mobile health research bus (February 2017 to June 2018), children who visited the bus were offered a DXA scan; scans were not available to those who were visited at home or in other community locations. Whole body scans were performed using the Lunar iDXA (GE Healthcare, Madison, WI, USA) by trained research nurses following study protocols describing how to perform daily checks and calibrations, the preparation of the study participant and the scanning procedure. Scans were delivered using the appropriate mode (paediatric or adult) and provided data on total and regional bone mineral content, FM and LM. For these analyses, we were interested total body fat percent (TBF%), and total, trunk, android, gynoid, arm and leg FM (kg). Regional boundaries were automatically demarcated using enCORE software version 14.0 and there were no instances of children not fitting within the scan field. The trunk region includes the neck, chest, abdominal and pelvic areas; the leg region includes the legs and lateral hip area; and the arms includes arms and shoulders. The android region is totally enclosed within the trunk and is defined the area between the ribs and the pelvis; the gynoid region includes the hips and upper thighs and overlaps both the leg and trunk regions.

### Anthropometric measurements

At the DXA scan appointment, height was measured to the nearest millimetre using the Leicester Height Measure and weight was measured to the nearest 0.1 kg using Tanita Body composition analyser SC-240. All measurements were taken by trained research assistants following standard operating procedures which described how to prepare and position the participants for height and weight measurements and how to record measurements accurately to reduce errors. BMI was calculated as weight/height ^2^ (kg/m ^2^) and converted to age- and sex-adjusted z-scores by comparison to the UK90 reference data ^25^. The proportion of children who were categorized as having overweight/obesity and obesity were defined as those with a z-score >85 ^th^ and 95 ^th^ centiles, respectively. TBF% was categorised into 15–24%, 25–34% and 35+%.

### Ethnicity

Ethnicity was self-reported by the mother when completing her baseline questionnaire in pregnancy and was used to define the ethnicity of her child based on UK Office of National Statistics guidance using the same classification as the 2001 UK census ^26^. We defined white ethnicity as those who identified as White British and White Other; SA was assigned to those of Pakistani, Indian and Bangladeshi heritage.

### Other measurements

To explore whether the DXA subsample included in these analyses were selected in a way that might introduce bias, we compared the following characteristics between the subgroup of white and SA children included in this study with white and SA mother and offspring dyads recruited to the full BiB cohort: maternal ethnicity, maternal age, maternal educational attainment, parity, IMD, smoking in pregnancy and early pregnancy BMI (based on height collected at the baseline questionnaire, and weight at pregnancy booking); and offspring sex, gestational age at birth, and birth weight (obtained from the hospital maternity system).

### Statistical analysis

P-values for differences in anthropometric and DXA measures between ethnic groups were calculated using chi-squared or t-tests. Pearson’s pairwise correlation was used to calculate the correlation between BMI and the DXA parameters. Age-adjusted linear regression was used to assess ethnic differences in TBF% and total and regional FM by BMI categories (healthy=BMI ≤85 ^th^ centile; overweight=>85 ^th^ to ≤95 ^th^ centile; obese>95 ^th^ centile). As FM outcomes were positively skewed, we used log values in the regression analysis. The results from these were exponentiated and are presented as ratios of geometric means per SD change in the outcome. Results are stratified by ethnicity and sex. All analyses were performed using STATA/SE v15 (StataCorp LP, College Station, TX, USA).

## Results

**Figure 1.**
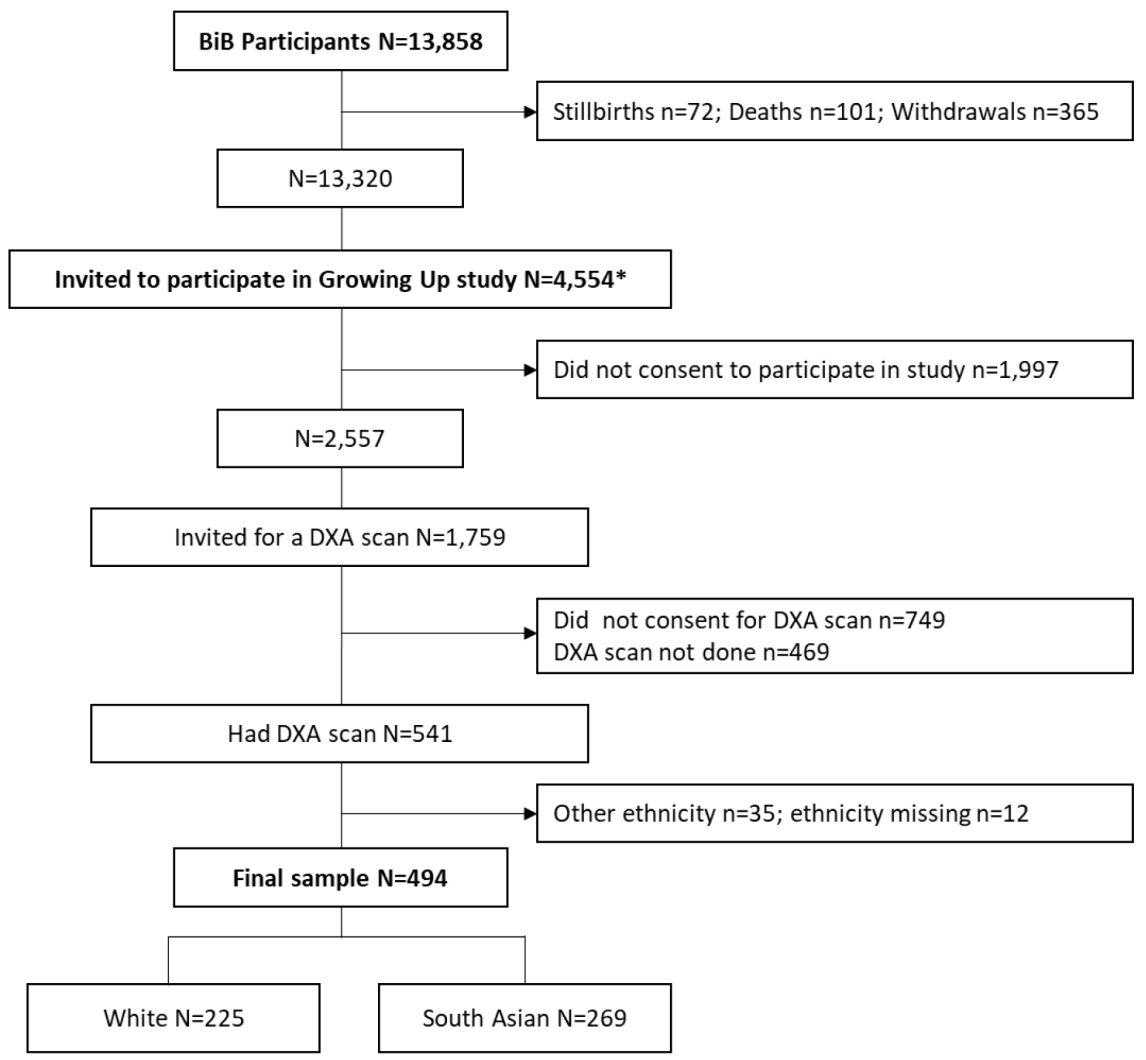
Flow chart of study sample. * Number invited between February 2017 and June 2018.

A flow chart of the study sample is presented in Figure 1. Between February 2017 and June 2018 (reflecting the loan period of the health research bus), 4,554 children aged ~9 years were invited to participate in the follow-up, and 2,557 consented. Of these, 1,759 agreed to visit the health research bus and were therefore offered a DXA scan, and 1,010 agreed. The scans were not performed on 469 of these children due to participant or research logistical and other issues, leaving a total of 541 children who received a scan. Of these, 225 were white, 269 were SA, 35 were of other minority ethnicity groups, and ethnicity data were missing for 12 children. As there were very few children of other ethnic minority groups, analyses of the DXA data were restricted to white and SA children only (see Figure 1).

Differences in key characteristics of those who had a DXA scan compared to the full BiB cohort are presented in Table 1. Within ethnic groups, the DXA sample comprised around 4% of the full cohort for white and SA participants, and 3% of other ethnic minority groups. Compared to the full BiB cohort, mothers of white children who had a DXA scan were older, more likely to live in an affluent area, be educated to degree level and less likely to have smoked during pregnancy. The birthweight and the proportion of males in other minority ethnic groups was greater in those who had a DXA scan compared to those who did not. There was more missing data on parity in white and SA children in the DXA sample, whereas participants in other ethnic groups who did not have a scan had a higher proportion of missing data for educational attainment and smoking during pregnancy (see Table 1).

**Table 1.** Comparison between the full BiB cohort ^1^ and the DXA scan sample. Values are mean (SD), median (IQR) or n (%).

	White		South Asian		Other ethnic group	
	Full cohort	DXA sample	Full cohort	DXA sample	Full cohort	DXA sample
	N=5,009	N=225 (4.3%)	N=6,249	N=269 (4.1%)	N=1,134	N=35 (3.0%)
**Maternal** **characteristics**						
Age (years)	26.7 (5.9)	30.3 (6.1)	28.0 (5.1)	28.4 (5.0)	28.0 (6.0)	29.7 (5.6)
Parity						
Nulliparous	2,276 (45.4)	100 (44.4)	1,926 (30.8)	67 (24.9)	433 (38.3)	11 (31.4)
Multiparous	2,461 (49.1)	104 (46.2)	3,991 (63.9)	172 (63.9)	598 (52.7)	21 (60.0)
Missing	272 (5.4)	21 (9.3)	332 (5.3)	30 (11.2)	103 (9.1)	3 (8.6)
IMD						
1 (most deprived)	2,405 (48.0)	89 (39.6)	4,634 (74.2)	201 (74.7)	806 (71.1)	23 (65.7)
2	1,216 (24.3)	65 (28.9)	1,252 (20.0)	50 (18.6)	205 (18.1)	6 (17.1)
3	716 (14.3)	30 (13.3)	215 (3.4)	14 (5.2)	71 (6.3)	6 (17.1)
4	419 (8.4)	30 (13.3)	105 (1.7)	4 (1.5)	31 (2.7)	0
5 (least deprived)	253 (5.1)	11 (4.9)	43 (0.7)	0	21 (1.9)	0
Education						
Less than degree level	3,588 (71.6)	112 (49.8)	3,867 (61.9)	155 (57.6)	408 (36.0)	17 (48.6)
Degree level	877 (17.5)	95 (42.2)	1,477 (23.6)	70 (26.0)	282 (24.9)	11 (31.4)
Missing	544 (10.9)	18 (8.0)	905 (14.5)	44 (16.4)	444 (39.2)	7 (20.0)
Smoking during pregnancy						
Yes	1,502 (30.0)	33 (14.7)	171 (2.7)	5 (1.9)	100 (8.8)	2 (5.7)
No	2,964 (59.2)	174 (77.3)	5,176 (82.8)	220 (81.8)	593 (52.3)	27 (77.1)
Missing	543 (10.8)	18 (8.0)	902 (14.4)	45 (16.4)	441 (38.9)	6 (17.1)
BMI (kg/m ^2^)	26.7 (6.0)	26.2 (5.1)	25.5 (5.4)	25.7 (4.8)	26.3 (5.6)	27.3 (6.1)
Missing, n (%)	862 (17.2)	37 (16.4)	1,247 (20.0)	63 (23.4)	514 (45.3)	8 (22.9)
**Child characteristics**						
Male	2,583 (51.6)	117 (52.0)	3,204 (51.3)	144 (53.5)	282 (51.3)	24 (68.6)
Gestational age (weeks)	40 (38, 40)	40 (38, 40)	39 (38, 40)	39 (38, 40)	39 (38, 40)	39 (38, 40)
Missing, n (%)	118 (2.3)	6 (2.7)	109 (1.7)	7 (2.6)	55 (4.9)	0
Birthweight (kg)	3.33 (0.57)	3.32 (0.61)	3.12 (0.54)	3.14 (0.54)	3.22 (0.56)	3.41 (0.52)
Missing, n (%)	131 (2.6)	3 (1.3)	333 (5.3)	11 (4.1)	54 (4.8)	1 (2.9)

DXA= Dual-energy X-ray absorptiometry; IMD=Index of multiple deprivation; BMI=body mass index

^1^Born in Bradford participants who did not have a DXA scan; excludes stillbirths, death, and withdrawals.

**Figure 2.**
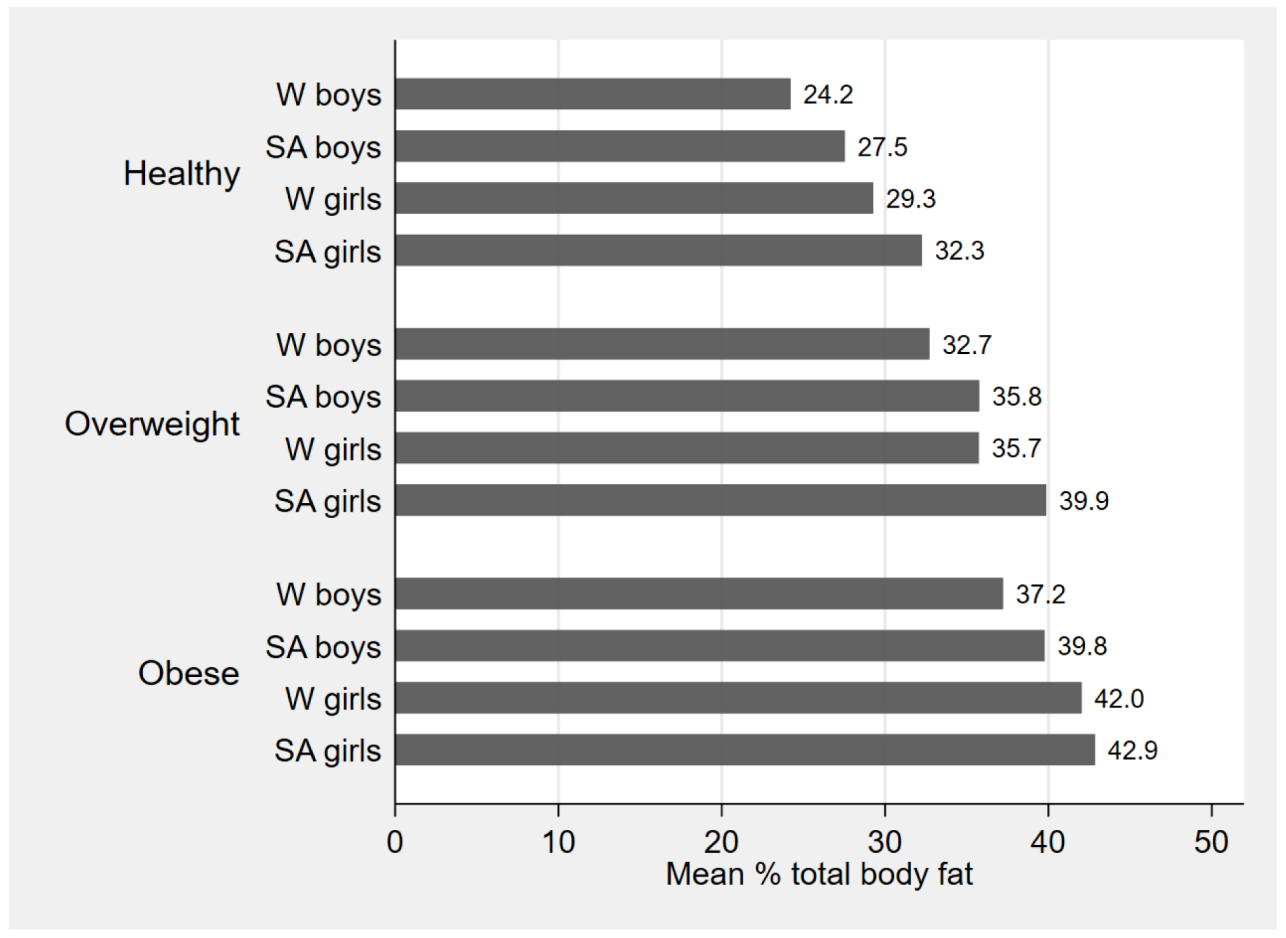
Mean % body fat by body mass index (BMI) category, stratified by sex and ethnic group. W=white; SA=South Asian

A summary of anthropometric and DXA measurements of the sample included in these analyses are presented in Table 2. SA boys were taller than white boys, on average, and a larger proportion of SA children were defined as living with overweight/obesity compared to their white counterparts (boys: 39.6% vs 29.1%; girls: 36.0% vs 27.8% of girls), but the mean BMI between the two groups was similar (boys: white 17.5 kg/m ^2^, SA 17.9 kg/m ^2^, P=0.317; girls: white 18.1 kg/m ^2^, SA 18.2 kg/m ^2^, P=0.817). The median difference in total FM in SA children compared to white children was 2.3 kg for boys and 2.1 kg in girls. SA children had on average a higher TBF% than white children, and the proportion with over 35% body fat was substantially greater: white boys 15.4%; SA boys 37.5%; white girls 37.0%; SA girls 52.8%. Figure 2 shows mean TBF% by BMI category in each sex and ethnic group and demonstrates higher TBF% in girls compared with boys and in SA compared with WB in all three categories of healthy weight, overweight and obesity. It also shows that in each sex and ethnic group TBF% was increased across the three BMI categories. BMI and all DXA measurements were highly correlated with each other in both ethnic groups ( Table 3).

**Table 2.** Participant characteristics by ethnicity and gender.

	Boys			Girls		
	White N=117	South Asian N=144	P-value	White N=108	South Asian N=125	P-value ^4^
Age (years) ^1^	9.1 (0.9)	9.1 (0.9)	0.981	9.2 (0.8)	9.2 (0.8)	0.923
**Anthropometric** **measurements**						
Height (cm) ^1^	136.9 (6.8)	138.9 (8.2)	0.042	137.7 (7.7)	138.4 (7.9)	0.489
Weight (kg) ^1^	33.2 (7.9)	35.1 (9.8)	0.089	35.3 (9.3)	35.3 (9.4)	0.682
BMI (kg/cm ^2^) ^1^	17.5 (3.1)	17.9 (3.6)	0.317	18.1 (3.4)	18.2 (3.7)	0.841
BMI z-score ^1^	0.33 (1.26)	0.40 (1.50)	0.680	0.37 (1.24)	0.33 (1.44)	0.833
BMI category ^2^			0.157			0.401
Healthy (<85 ^th^ centile)	83 (70.9)	87 (60.4)		78 (72.2)	80 (64.0)	
Overweight (≥85 ^th^ – 94 ^th^ centile)	11 (9.4)	23 (16.0)		12 (11.1)	19 (15.2)	
Obese (≥95 ^th^ centile)	23 (19.7)	34 (23.6)		18 (16.7)	26 (20.8)	
**DXA data**						
Mean absolute TBF% (SD) ^1^	27.6 (7.1)	31.7 (7.3)	<0.001	32.1 (7.1)	35.7 (6.5)	<0.001
Avg. TBF% category ^2^						
15–24%	49 (41.9)	32 (22.2)	<0.001	21 (19.4)	7 (5.6)	0.002
25–34%	50 (42.7)	58 (40.3)		47 (43.5)	52 (41.6)	
35+%	18 (15.4)	54 (37.5)		40 (37.0)	66 (52.8)	
Total body FM ^3^	7.6 (5.4, 11.1)	9.9 (6.4, 14.6)	0.001	9.4 (6.8, 13.9)	11.5 (8.2)	0.012
Trunk FM ^3^	2.7 (1.7, 4.4)	3.8 (2.1, 6.1)	0.001	3.6 (2.4, 5.8)	4.7 (3.1, 7.2)	0.003
Android FM ^3^	0.3 (0.2, 0.6)	0.5 (0.2, 0.9)	<0.001	0.4 (0.3, 0.8)	0.6 (0.4, 1.1)	0.002
Gynoid FM ^3^	1.3 (0.9, 1.8)	1.6 (1.1, 2.3)	0.001	1.7 (1.2, 2.5)	2.0 (1.4, 2.6)	0.010
Android/gynoid ratio	0.27 (0.10)	0.31 (0.12)	<0.001	0.30 (0.11)	0.35 (0.11)	<0.001
Arms FM ^3^	0.9 (0.6, 1.3)	1.2 (0.8, 1.7)	0.006	1.2 (0.8, 1.6)	1.3 (1.0, 1.7)	0.106
Legs FM ^3^	3.4 (2.3, 4.5)	4.2 (2.7, 5.8)	<0.001	4.1 (3.0)	4.7 (3.5, 6.0)	0.027
Lean mass (kg) ^1^	23.7 (3.8)	23.2 (4.4)	0.394	23.0 (3.9)	22.0 (4.0)	0.063
Bone mineral content (kg) ^1^	1.12 (0.18)	1.20 (0.22)	0.489	1.17 (0.19)	1.16 (0.20)	0.549

DXA= Dual-energy X-ray absorptiometry; BMI=body mass index; TBF% = total body fat percentage; FM=fat mass (kg). ^1^Mean (SD). ^2^N (%). ^3^Median (IQR). ^4^Calculated using t-tests, chi-squared tests or Mann-Whitney tests, as appropriate.

**Table 3.** Correlation matrix of BMI with DXA parameters in white (not italic) and South Asian (italic) children.

	BMI	Fat mass (kg)					
		Total body	Trunk	Android	Gynoid	Arms	Legs
BMI	1.00						
Total body	0.942 *0.939*	1.00					
Trunk	0.912 *0.918*	0.980 *0.984*	1.00				
Android	0.921 *0.927*	0.975 *0.983*	0.990 *0.990*	1.00			
Gynoid	0.894 *0.919*	0.974 *0.983*	0.967 *0.960*	0.953 *0.954*	1.00		
Arms	0.937 *0.918*	0.974 *0.977*	0.931 *0.952*	0.935 *0.952*	0.935 *0.958*	1.00	
Legs	0.922 *0.913*	0.970 *0.965*	0.903 *0.904*	0.903 *0.916*	0.932 *0.964*	0.962 *0.945*	1.00

DXA= Dual-energy X-ray absorptiometry; BMI=body mass index

**Figure 3.**
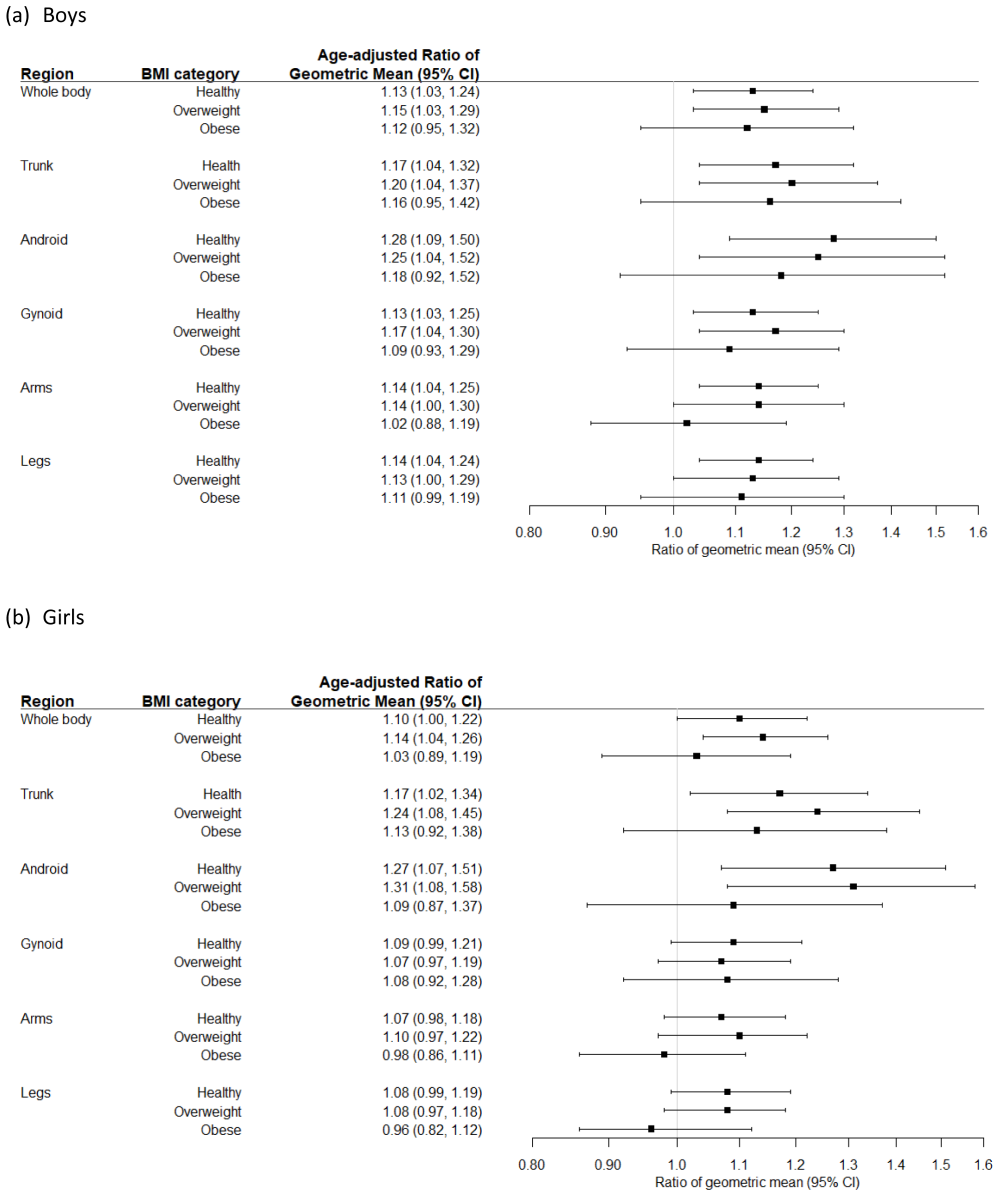
Age-adjusted ratios of geometric means (95% CI) for total and compartmental fat mass comparing South Asian to white children, in each body mass index (BMI) category.

Figure 3 shows the age-adjusted ratios of geometric means of total and compartmental FM in SA compared to white children, by BMI category. Healthy- and overweight SA boys and girls had greater total and regional FM compared to white boys and girls in the same BMI category, with the greatest differences observed in the android region: in healthy weight SA boys and girls this was 28% and 27% higher respectively compared to white children, and in overweight children android FM was 25% higher in SA boys and 31% higher in SA girls. Healthy or overweight SA boys also had 14% greater adiposity in their arms and legs compared to white boys, but there was no difference between ethnic group in girls. No ethnic differences in total and compartmental FM were observed in children living with obesity.

## Discussion

We have demonstrated some evidence of ethnic differences in the distribution and amount of directly measured total and regional adiposity, and their relationship with established categories of BMI. Whilst no statistically significant difference in BMI was observed between white and SA children, on average SA children had higher TBF%, with over one-third of boys and half of girls having >35% TBF. Age adjusted relative differences (ratios of geometric means) in FM in general support higher total and truncal fat mass in SA compared with white children. A previous study of the BiB cohort at 4–5 years found that triceps skinfolds – an indicator of peripheral fat – was lower in SA boys and girls compared to white children ^27^; this contrasts with the higher arm and leg FM observed in SA boys (but not girls) in the present study, which may indicate that in addition to greater central adiposity, SA boys may also be susceptible to increased peripheral adiposity as they grow.

Our findings of ethnic differences in TBF% fat but not BMI in white and SA children is consistent with other UK studies which measured adiposity using precise techniques ^15, 22^ thereby adding further evidence that using BMI alone may underestimate adiposity in SA children. One of these studies, which included 339 SA and 654 white children between the ages of 6 to 18 years, reported the proportion of children in three different categories of TBF%: less than 15%, 15–25% and more than 25%, and found that a higher proportion of SA children were in the highest category ^22^. When we used these definitions, we found there were no children in the lowest category, meaning that we needed to apply higher thresholds in our analyses. The difference in the proportion of SA compared to white children in the highest category was far greater than the previous study. There is a 15–20-year difference between the data collection of the sample in that study and ours and our results possibly demonstrate a worrying trend in the increasing adiposity in children, particularly in UK-born SA children. This is reflected in the UK National Child Measurement Programme (NCMP) figures for 4–5- and 10–11-year olds over the past 15 years ^28^, which show a temporal increase in the proportion of children classified as obese: between 2006/07 and 2019/20, the percentage of 10–11 year olds living with obesity has increased from 16% to 19% in white children, and from 21% to 25% in SA children.

Only one other study exploring the associations of BMI with directly measured regional fat mass in UK adolescents was identified ^8^. It included 2,840 children, and reported similar associations between BMI and DXA FM with cardiometabolic risk factors as other studies in that it was abdominal fatness that was the primary driver of cardiometabolic dysfunction. Again, this study was conducted in a predominantly white European population, so the increased risk of cardiometabolic disease in other ethnic groups who display greater central adiposity remains unknown. We also found that SA children had greater android and gynoid FM, and higher android/gynoid ratio compared to white children, though it is acknowledged that the confidence intervals are wide due to the sample size. A handful of studies have found that android/gynoid ratio was more strongly associated with cardiometabolic health in children ^18– 20^. This appears to persist into adulthood, with co-mingling of android and gynoid adiposities being associated with greater cardiometabolic risk than android or gynoid adiposity alone ^17^.

Our findings suggest that SA children may be at increased risk of adipose-related morbidity even when they are considered a healthy weight according to their BMI, and that their risk when overweight is potentially far greater than that of white children. SA children who are defined as healthy or overweight could therefore be more metabolically unhealthy than white children in the same BMI category.

### Strengths and limitations

We were able to report total and regional FM in a bi-ethnic sample measured using DXA, which is an extremely accurate method of measuring body composition. However, a limitation is that DXA cannot distinguish between visceral and subcutaneous fat, and it is the former that appears to be the primary driver of cardiometabolic dysfunction.

There were some differences between the sample in the current study and the wider BiB cohort. Whilst most characteristics were similar in SA with DXA scans and the full original cohort, white mothers whose children attended a DXA scan tended to be older, educated to degree level and less likely to smoke during pregnancy or reside in deprived areas compared to the white population in the full BiB cohort. As higher levels of obesity are associated with increasing deprivation ^29^, the sample in the current study may be biased in that these children were less likely to be overweight/obese than those in the full cohort. However, whilst SA children in this sample were mostly disadvantaged, two thirds of white children were also from the two most deprived IMDs. The observed differences between the full cohort and the DXA sample may also be partly driven by the proximity of participants residence to the location of the mobile unit where the DXA scans were conducted, though it was, at various times, sited at four different areas of the city to widen the catchment area. We found no difference in measurements of height, weight, or BMI z-scores in the DXA sample compared to the other participants of the follow-up study, and we see no reason why levels of adiposity would be systematically different.

We acknowledge that our study sample is small; we did not adjust for any confounders or factors related to selection into the DXA study, beyond age and were not able to detect differences between BMI categories or sex, with all estimates having wide confidence intervals. Thus, our findings need to be treated with some caution unless replicated in larger studies with greater ability to adjust for potential confounders and explore possible selection bias. Further, our sample, although representative of the region it was drawn from, may not reflect the same characteristics found in other multi-ethnic areas, including those with SA populations that are not predominantly of Pakistani heritage, as in our study. Finally, as this is a cross-sectional study, we were not able to observe any changes in regional FM throughout childhood and adolescence.

To conclude, we have demonstrated that although there were no differences in BMI between white and SA children, SA children had greater total and regional FM compared to white children. Further, although the proportion of SA children classified as overweight and obese was higher than in white children, the greatest differences in total and regional FM between the two ethnic groups were observed in children of a healthy weight and who were overweight; no differences were apparent in children living with obesity. The implications of this are that SA children may be at greater risk of cardiometabolic disease compared to white children with a similar BMI, and failure to recognise this at an early age may delay appropriate action, such as referrals to obesity interventions. Given the size of our study and lack of adjustment, findings need to be replicated in a larger study with power to adjust for confounders. Further research on the associations of these differences with cardiometabolic risk, and the use of ethnic-adjusted BMI thresholds where indicated to better identify children at risk, is required.

## Data availability

The data referenced by this article are under copyright with the following copyright statement: Copyright: ï¿½ 2021 Santorelli G et al.

Data associated with the article are available under the terms of the Creative Commons Zero "No rights reserved" data waiver (CC0 1.0 Public domain dedication). 

### Underlying data

Harvard Dataverse: Replication Data for: Differences in total and regional body fat and their association with BMI in UK-born White and South Asian Children: Findings from the Born in Bradford birth cohort. https://doi.org/10.7910/DVN/WEO4XI ^30^.

This project contains the following underlying data:

- Raw dataset including demographic and outcome variables of the sample

Data are available under the terms of the Creative Commons Zero "No rights reserved" data waiver (CC0 1.0 Public domain dedication).
