# Supplementary material for: Differences in total and regional body fat and their association with BMI in UK-born White and South Asian children: findings from the Born in Bradford birth cohort
Source: Wellcome Open Res. 2022 Apr 1;6:65. Originally published 2021 Mar 26. [Version 3] doi: 10.12688/wellcomeopenres.16659.3 (PMC10611948; doi:10.12688/wellcomeopenres.16659.3)
Supplement: Peer review history

# Reviewer report: Reviewer response for version 3

**DOI:** 10.21956/wellcomeopenres.19736.r68170

**Published:** 2023-10-27

**Authors:** Rabab Alkutbe

**Affiliations:** 1 Research and Innovation Department, Saudi Patient Safety Center, Riyadh, Saudi Arabia

This is a detailed study attempting to determine any differences in total FM that objectively measured using DXA, and the association between the total FM and the BMI category, in UK-born white and SA-origin children aged 9 years. Although this study has many strengths, I think the below points would add value to the study:

- I think there is excellent data on the maternal characteristics' from the respondents. It would be interesting to see how these maternal characteristics could affect the children FM and body compositions.
- The author may need to apprise the socioeconomic factors by exploring more the correlation between children with obesity/OW and maternal BMI, education level, and smoking etc.

Is the work clearly and accurately presented and does it cite the current literature?

Yes

If applicable, is the statistical analysis and its interpretation appropriate?

Yes

Are all the source data underlying the results available to ensure full reproducibility?

Yes

Is the study design appropriate and is the work technically sound?

Yes

Are the conclusions drawn adequately supported by the results?

Yes

Are sufficient details of methods and analysis provided to allow replication by others?

Yes

Reviewer Expertise:

Body composition, Children, Obesity, public health, Patient Safety

I confirm that I have read this submission and believe that I have an appropriate level of expertise to confirm that it is of an acceptable scientific standard.

# Reviewer report: Reviewer response for version 3

**DOI:** 10.21956/wellcomeopenres.19736.r67099

**Published:** 2023-10-24

**Authors:** Andriana Anagnostopoulou

**Affiliations:** 1 Aghia Sophia Children's Hospital, Athens, Greece

This is an interesting article.

 This is a cross sectional study which followed up children of a birth cohort of mothers.It combined anthromporphic measurements with infomrmation from dxa scan.

 Some revisions suggested:

1. Did authors calculate selection bias, which they mentioned in the weaknesses part. How did they reach the sample power?
2. The BW of the SA cohort was lower than the white counterparts, as has been reported. Did they stratify according to the birth weight?
3. The authors write "Although mean BMI was similar, compared to white children, the proportion of SA children who were overweight or obese was ~10% higher, and the proportion with > 35% total body fat (TBF) was 22% and 16% higher in boys and girls respectively.".How did they reach that conclusion? Did they include other anthropometric parameters such as waist to hip ratio? That statement is not fully explained.

Is the work clearly and accurately presented and does it cite the current literature?

Yes

If applicable, is the statistical analysis and its interpretation appropriate?

Yes

Are all the source data underlying the results available to ensure full reproducibility?

Yes

Is the study design appropriate and is the work technically sound?

Yes

Are the conclusions drawn adequately supported by the results?

Yes

Are sufficient details of methods and analysis provided to allow replication by others?

Yes

Reviewer Expertise:

Paediatric cardiologist

I confirm that I have read this submission and believe that I have an appropriate level of expertise to confirm that it is of an acceptable scientific standard, however I have significant reservations, as outlined above.

# Reviewer report: Reviewer response for version 3

**DOI:** 10.21956/wellcomeopenres.19736.r49621

**Published:** 2022-05-18

**Authors:** Ken K. Ong

**Affiliations:** 1 MRC Epidemiology Unit & Department of Paediatrics, Institute of Metabolic Science, School of Clinical Medicine, University of Cambridge, Cambridge, UK

The authors have responded appropriately to my comments.

 Apologies for not spotting this at the previous review, but in the added Figure 2 the vertical lines indicating thresholds for overweight and obesity at age 9 years are differently situated between the two panels - these should be identical. On redrawing these figures, it would be much easier to compare the distributions if one were placed above the other (instead of side-by-side).

Is the work clearly and accurately presented and does it cite the current literature?

Yes

If applicable, is the statistical analysis and its interpretation appropriate?

Partly

Are all the source data underlying the results available to ensure full reproducibility?

Yes

Is the study design appropriate and is the work technically sound?

Yes

Are the conclusions drawn adequately supported by the results?

Partly

Are sufficient details of methods and analysis provided to allow replication by others?

Yes

Reviewer Expertise:

Epidemiology, child growth and body composition

I confirm that I have read this submission and believe that I have an appropriate level of expertise to confirm that it is of an acceptable scientific standard.

# Reviewer report: Reviewer response for version 2

**DOI:** 10.21956/wellcomeopenres.19190.r46932

**Published:** 2022-01-04

**Authors:** Ken K. Ong

**Affiliations:** 1 MRC Epidemiology Unit & Department of Paediatrics, Institute of Metabolic Science, School of Clinical Medicine, University of Cambridge, Cambridge, UK

I am happy that the authors have addressed my previous comments. I have added a couple more comments that arise from similar points. These comments are straightforward but do need to be addressed:

**Abstract results:**

- "the proportion of SA children who were overweight or obese was ~20% higher". - This seems to be incorrect. The differences were 10.5% and 8.2% in boys and girls.

**Strengths & Limitations Para 3:**

- "We acknowledge that our study sample is small". - Add for clarification 'and differences in overweight and obesity status did not reach statistical significance'.
- "It is therefore unlikely that early menstruation is a factor in our population". - Replace 'early menstruation' with 'early puberty timing'. Menstruation is only a (relatively late) marker of puberty timing and is not in itself a direct factor in body growth and composition.

Is the work clearly and accurately presented and does it cite the current literature?

Yes

If applicable, is the statistical analysis and its interpretation appropriate?

Partly

Are all the source data underlying the results available to ensure full reproducibility?

Yes

Is the study design appropriate and is the work technically sound?

Yes

Are the conclusions drawn adequately supported by the results?

Partly

Are sufficient details of methods and analysis provided to allow replication by others?

Yes

Reviewer Expertise:

Epidemiology, child growth and body composition

I confirm that I have read this submission and believe that I have an appropriate level of expertise to confirm that it is of an acceptable scientific standard, however I have significant reservations, as outlined above.

# Response

**Published:** 2022-03-30

**Authors:** Gillian Santorelli

**Affiliations:** Bradford Hospitals National Health Service Trust, UK

Many thanks to the reviewer for highlighting the error in the abstract, which has now been corrected. The suggested amendments to the text have also been made.

# Reviewer report: Reviewer response for version 1

**DOI:** 10.21956/wellcomeopenres.18370.r43559

**Published:** 2021-05-24

**Authors:** Ken K. Ong

**Affiliations:** 1 MRC Epidemiology Unit & Department of Paediatrics, Institute of Metabolic Science, School of Clinical Medicine, University of Cambridge, Cambridge, UK

This cross sectional analysis of 9 year old children in the Born in Bradford cohort reports that despite having the same level of BMI, South Asian children had greater total body and central fat mass than white children.

1. However part of this difference may be because South Asian children were taller at age 9 years, especially the boys. This will contribute to having higher absolute fat mass, whereas BMI is (mostly) corrected for height. All fat mass comparisons should be adjusted for height.
2. Other UK cohorts have described that early puberty is 3x more likely in SA girls than white girls https://pubmed.ncbi.nlm.nih.gov/27672135/. ^1^ As puberty leads to accelerated fat gains in girls, this is another potential confounder. Do they have information on puberty status at this timepoint? If not, this should be discussed as a limitation. 
3. South Asian children had a similar mean BMI to white children, yet SA have a higher prevalence of OW & OB. How can they be sure then that BMI is adequately controlled for? Discussion (line 2-3, also 1st line of the concluding paragraph) "no statistically significant difference in BMI was observed" - clarify that this refers to 'mean BMI' but that there were differences in BMI category.
4. Introduction (para 1): "gynoid fat (i.e. in the gluteofemoral region), which is subcutaneous…" This line confuses different ways to partition types of fat. Central:peripheral partitioning is quite different to visceral:subcutaneous and even intramuscular.
5. Introduction (para 2): "(BMI) is highly correlated with both (fat mass and lean mass)" - BMI is a good proxy marker of adiposity because, at least in adults, its correlations with fat mass are stronger than those with lean mass (e.g. see https://pubmed.ncbi.nlm.nih.gov/28096530/). ^2^ However, this is not necessarily true in children.
6. Methods: "Ethnicity was self-reported by the mother…in pregnancy" i.e. only by one parent. How big a limitation is this as a proxy for the child's ethnicity? What is the prevalence of mixed marriages in this setting?
7. Methods: The included sample here comprises only 4% of the full cohort for white and SA participants. While this was mostly for logistical issues, e.g. having visits within a certain timescale, there was a substantial drop from visits to DXA scans: "1,759 agreed to visit the health research bus and were therefore offered a DXA scan, and (only) 1,010 agreed". This is surprising as it seems only a small step in practice to have a DXA once you visit the research bus. What were the reasons for refusing DXA and more importantly did this proportion differ by ethnicity and BMI category?

Some typo errors:

  

1. Methods - Anthropometry: Definition of OW and obesity should read "defined as those with a BMI >85th or >95th centile" i.e. don't confuse z-scores and percentiles
2. Statistical analyses: "presented as … per SD change in the outcome", I think should be 'in the exposure' (not the outcome)
3. Table 2: "Mean absolute TBF%" - 'absolute' is unnecessary. What is "Avg. TBF%"? (simply 'TBF%'?)

Is the work clearly and accurately presented and does it cite the current literature?

Yes

If applicable, is the statistical analysis and its interpretation appropriate?

Partly

Are all the source data underlying the results available to ensure full reproducibility?

Yes

Is the study design appropriate and is the work technically sound?

Yes

Are the conclusions drawn adequately supported by the results?

Partly

Are sufficient details of methods and analysis provided to allow replication by others?

Yes

Reviewer Expertise:

Epidemiology, child growth and body composition

I confirm that I have read this submission and believe that I have an appropriate level of expertise to confirm that it is of an acceptable scientific standard, however I have significant reservations, as outlined above.

## References

[1] : Early puberty in 11-year-old girls: Millennium Cohort Study findings. *Arch Dis Child* .2017;102(3) : 10.1136/archdischild-2016-310475 232-237 10.1136/archdischild-2016-310475 27672135PMC5339561

[2] : Associations between body mass index-related genetic variants and adult body composition: The Fenland cohort study. *Int J Obes (Lond)* .41(4) : 10.1038/ijo.2017.11 613-619 10.1038/ijo.2017.11 28096530PMC5382973
